# Effects of Er:YAG and Diode Laser Irradiation on Dental Pulp Cells and Tissues

**DOI:** 10.3390/ijms19082429

**Published:** 2018-08-17

**Authors:** Shunjiro Yamakawa, Takahiko Niwa, Takeo Karakida, Kazuyuki Kobayashi, Ryuji Yamamoto, Risako Chiba, Yasuo Yamakoshi, Noriyasu Hosoya

**Affiliations:** 1 Department of Endodontology, School of Dental Medicine, Tsurumi University, 2-1-3 Tsurumi, Tsurumi-ku, Yokohama 230-8501, Japan; 2711012@stu.tsurumi-u.ac.jp (S.Y.); hosoya-n@tsurumi-u.ac.jp (N.H.); 2 Department of Periodontology, School of Dental Medicine, Tsurumi University, 2-1-3 Tsurumi, Tsurumi-ku, Yokohama 230-8501, Japan; niwa-takahiko@tsurumi-u.ac.jp; 3 Department of Biochemistry and Molecular Biology, School of Dental Medicine, Tsurumi University, 2-1-3 Tsurumi, Tsurumi-ku, Yokohama 230-8501, Japan; karakida-t@tsurumi-u.ac.jp (T.K.); yamamoto-rj@tsurumi-u.ac.jp (R.Y.); chiba-r@tsurumi-u.ac.jp (R.C.); 4 Department of Dental Hygiene, Tsurumi Junior College, 2-1-3 Tsurumi, Tsurumi-ku, Yokohama 230-8501, Japan; kobayashi-kz@tsurumi-u.ac.jp

**Keywords:** Er:YAG laser, diode laser, dental pulp cell, apoptosis, gene, matrix metalloprotease, transforming growth factor-beta

## Abstract

Vital pulp therapy (VPT) is to preserve the nerve and maintain healthy dental pulp tissue. Laser irradiation (LI) is beneficial for VPT. Understanding how LI affects dental pulp cells and tissues is necessary to elucidate the mechanism of reparative dentin and dentin regeneration. Here, we show how Er:YAG-LI and diode-LI modulated cell proliferation, apoptosis, gene expression, protease activation, and mineralization induction in dental pulp cells and tissues using cell culture, immunohistochemical, genetic, and protein analysis techniques. Both LIs promoted proliferation in porcine dental pulp-derived cell lines (PPU-7), although the cell growth rate between the LIs was different. In addition to proliferation, both LIs also caused apoptosis; however, the apoptotic index for Er:YAG-LI was higher than that for diode-LI. The mRNA level of odontoblastic gene markers—two dentin sialophosphoprotein splicing variants and matrix metalloprotease (MMP)20 were enhanced by diode-LI, whereas MMP2 was increased by Er:YAG-LI. Both LIs enhanced alkaline phosphatase activity, suggesting that they may help induce PPU-7 differentiation into odontoblast-like cells. In terms of mineralization induction, the LIs were not significantly different, although their cell reactivity was likely different. Both LIs activated four MMPs in porcine dental pulp tissues. We helped elucidate how reparative dentin is formed during laser treatments.

## 1. Introduction

Vital pulp therapy (VPT) encompasses clinical interventions to preserve the nerve as much as possible, and to maintain healthy pulp tissue. VPT is carried out to reverse pulpal injury, and promote continued root development and apical closure, and to accomplish root canal therapy regarding dental literature. Phototherapy with various lasers has been widely accepted for its analgesic effects and promotion of wound healing to treat stomatitis, dentin hypersensitivity, temporomandibular joint disorder, periodontal disease, and caries [1,2,3,4]. In root canal therapy, the laser contributes to sterilization, disinfection, drying, and transpiration of infected dentin in root canal. The laser that penetrates into the tissue is used for coagulation of the exposed pulp, thereby creating the biological bases for the formation of reparative dentin.

Erbium-doped yttrium aluminum garnet (Er:YAG) lasers have numerous applications in a wide range of not only medical, but also dental fields. Er:YAG lasers have high water absorbency compared to CO_2_ and neodymium-doped yttrium aluminum garnet (Nd:YAG) lasers [5]. Due to this characteristic, Er:YAG lasers are well absorbed in biological tissues containing water, and used for various treatments, such as cementum transpiration of the root surface, tartar, bone tissue, and gingival soft tissue [6,7,8,9,10,11]. Unlike Er:YAG lasers, which are absorbed on the tissue surface, diode lasers are transmitted to internal tissue and used for incision, hemostatic coagulation, and pain relief for intraoral soft tissue [12,13].

Research in vitro has demonstrated that laser irradiation (LI) promotes cell migration and proliferation [14,15], mitochondrial respiration [16,17,18], protein synthesis [14,19] and bone formation [20]. Dental pulp stem cells (DPSCs) have a mesenchymal stem cell (MSC) phenotype, and the potential to differentiate into multiple cell types, including odontoblasts, osteoblasts, chondrocytes, cardiomyocytes, hepatocytes, neuron, liver cells, and pancreatic islet β cells. In addition, DPSCs are also used for induced pluripotent stem (iPS) cell creation and priming. DPSCs are generally assumed to differentiate into each cell type by expressing marker genes, enhancing alkaline phosphatase (ALP) activity and forming precipitated nodules. In dental research, dental pulp tissues containing DPSCs have been used to establish a number of odontoblastic cell lines from rat [21,22], mouse [23], cow [24], pig [25], and human [26,27]. Pulp capping materials, such as calcium hydroxide and mineral trioxide aggregate, induce reparative dentin formation by causing the release of growth factors from the dentin matrix [28]. A variety of factors, such as bone morphogenetic protein (BMP) [29,30], fibroblast growth factors [31], and transforming growth factor beta (TGF-β), regulate dental pulp cell differentiation [32,33] and odontoblastic differentiation, many of which are associated with ectoderm–mesenchyme molecular interactions. TGF-β released from the dentin matrix and extracellular matrix molecules can induce the differentiation of progenitor/stem cells into odontoblast-like cells [28], but the dynamics and mechanisms of how LI affects DPSC odontoblastic differentiation remain unknown. In addition, little is known about how LI elicits changes in dental pulp tissues.

Here, we report the effects of Er:YAG-LI and diode-LI on proliferation, apoptosis, gene expression, mineralization induction, and protease activation in dental pulp cells and tissues.

## 2. Results

### 2.1. Cell Proliferation Rate of PPU-7

**Figure 1:**
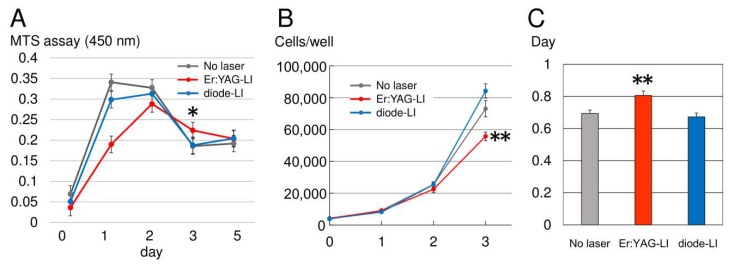
Effect of laser irradiation (LI) on PPU-7 proliferation incubated for different periods. (**A**) MTS assay. PPU-7 after Er:YAG-LI (red), diode-LI (blue), and without LI (grey) on day 0, 1, 2, 3, and 5, were cultured at a final volume of 120 μL/well for 1 h at 37 °C. MTS reagent was added, and an absorbance of 450 nm was recorded using a microplate reader (*n* = 10 tests per sample). Values are the mean ± standard error (* *p* < 0.01, Steel’s test). (**B**) The number of PPU-7 cells. PPU-7 cells were counted on day 0, 1, 2, and 3 after laser irradiation (** *p* < 0.05, Steel’s test). (**C**) Cell population doubling level against days after laser irradiation. Data are means ± standard error (** *p* < 0.05, Steel’s test).

**Figure A1:**
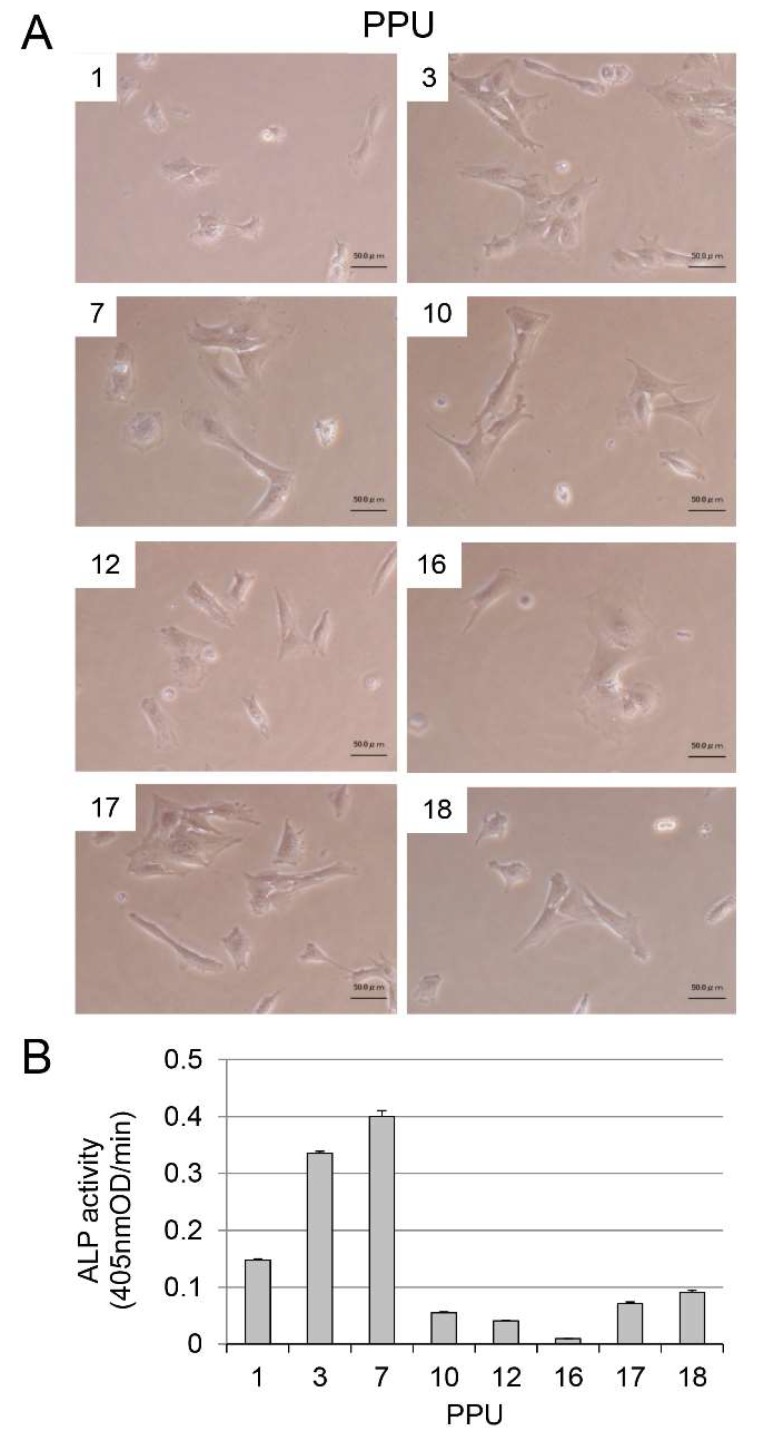
Morphology and inherent alkaline phosphatase (ALP) activity of porcine dental pulp-derived cell lines. (**A**) Eight dental pulp-derived cell lines (PPU-1, -3, -7, -10, -12, -16, -17, and -18) are shown. Bars = 50 μm. (**B**) Inherent ALP activity of the respective cell lines. Data bars are means ± standard error (SEM) of 6 culture wells.

**Figure A2:**
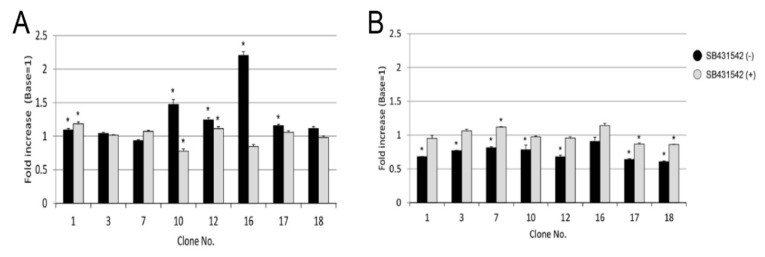
Effect of BMP2 and TGF-β1 on inherent ALP activity of porcine dental pulp-derived cell lines. Effect of (**A**) BMP2 with or without LDN-193189, and (**B**) TGF-β1 with or without SB431542. Values of inherent ALP activity are indicated as the fold increase, when the activity of the control was 1. Data are means ± standard error (SEM) of 6 culture wells (* *p* < 0.05).

**Figure A3:**
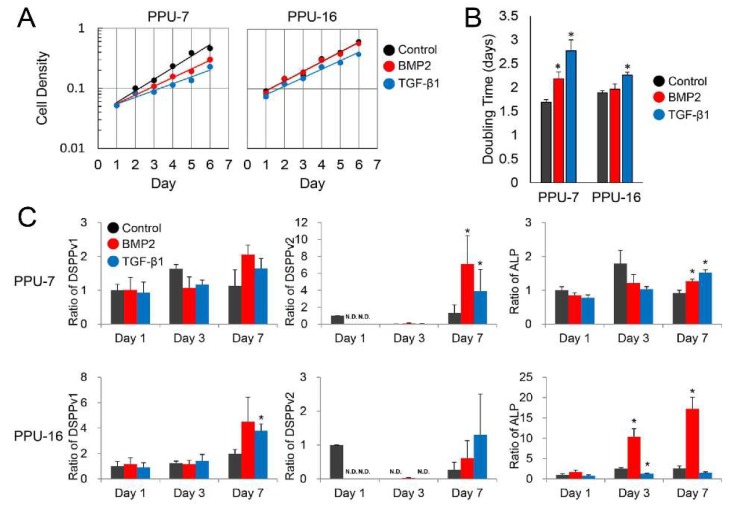
Effect of BMP2 and TGF-β1 on proliferation and gene expression of PPU-7 and PPU-16 cell lines. (**A**) Cell density plotted on a semi-logarithmic scale was used to judge the logarithmic growth phase by linearity, which was determined, in turn, every day for 6 days. (**B**) Cell population doubling level against days in culture following transfection. Data are means ± standard error (SEM) of 6 culture wells. BMP2 and TGF-β1 in PPU-7 or BMP2 in PPU-16 significantly required time (* *p* < 0.05). (**C**) The mRNA expression by qPCR analysis of DSPP-variant 1 (DSPP-v1, left), DSPP-variant 2 (DSPP-v2, middle), and ALP (right). Each ratio was normalized to glyceraldehyde-3-phosphate dehydrogenase (GAPDH) as a reference gene, and the relative quantification data of DSPP-v1, DSPP-v2, and ALP in PPU-7 or PPU-16 were generated on the basis of a mathematical model for relative quantification in a qPCR system (*n* = 6 PPU-7 or PPU-16). All expression levels are indicated as the increasing or decreasing ratio, when the mRNA level of control (Day 1) was 1. Data are means ± standard error (SEM) (* *p* < 0.05). N.D.: not determined.

We established a porcine dental pulp-derived cell line (PPU-7) (see Figure A1 and Figure A2 in Appendix A.1, Figure A3 in Appendix A.2 and Appendix A.3 in Appendix A) and investigated the effects of Er:YAG-LI and diode-LI on proliferation (Figure 1). In MTS assay (Figure 1A), the proliferation rate under diode-LI was the same as that in the control (no LI), and the cells reached confluence in the first day. By contrast, the proliferation rate under Er:YAG-LI in the first day was approximately half of that under diode-LI, and the cells reached confluence in the second day. On the third day, cell proliferation under the diode laser rapidly decreased at the same rate as the control, whereas the Er:YAG laser-treated cells showed a gradual downward trend. The decrease in proliferative rates for Er:YAG-LI and diode-LI were the same by the fifth day. The number of PPU-7 was counted on day 0, 1, 2, and 3 after laser irradiation (Figure 1B). The cell number after Er:YAG-LI and diode-LI was almost the same as that in the control without laser irradiation in the first day. However, the cell number of Er:YAG-LI began to decrease in the second day, and it was lower than control (1.42-fold) and diode-LI (1.51-fold) in the third day. Compared with control and diode-LI, the cell population doubling level of PPU-7 until day 3 required time with Er:YAG-LI (Figure 1C).

### 2.2. Apoptosis of PPU-7

**Figure 2:**
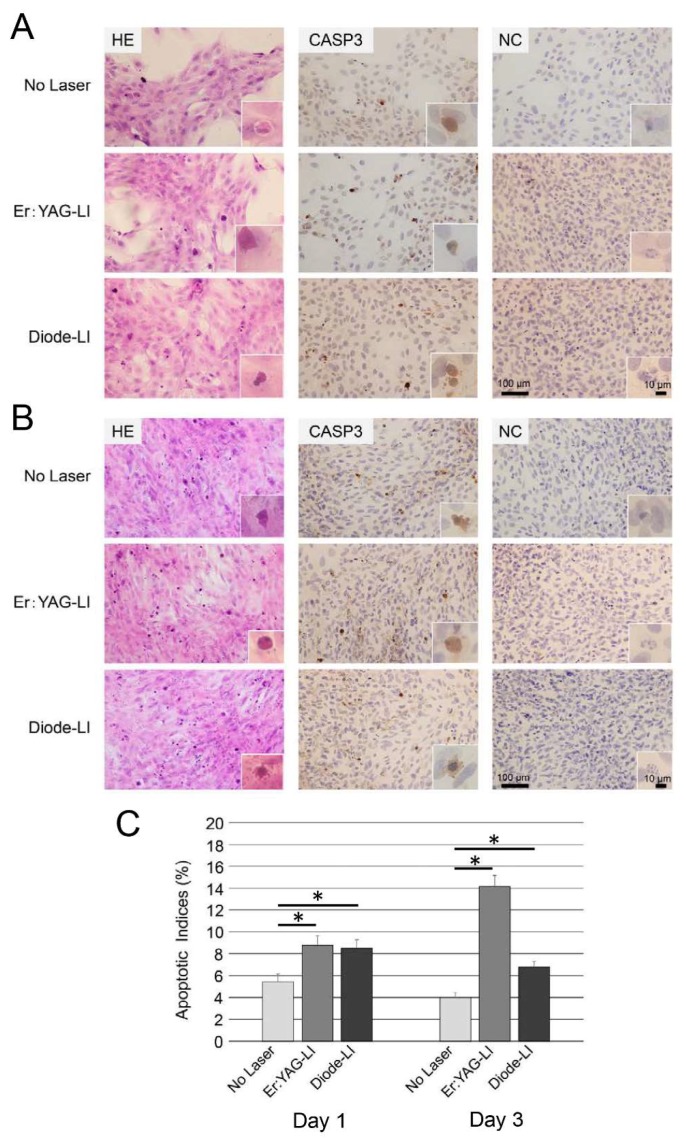
Effect of LI on apoptosis in PPU-7. Immunohistochemical detection of apoptosis in PPU-7 on (**A**) day 1 and (**B**) day 3 following LI. Eosinophilic apoptotic bodies in hematoxylin-eosin-stained PPU-7 detected by transmitted-light microscopy (HE) (magnification: 400×). Apoptotic bodies in PPU-7 stained by cleaved caspase-3 antibody (CASP3); the control was processed without primary antibody (NC). The images are high magnification of the area boxed in the Figure. (**C**) Apoptotic indices in PPU-7 with or without laser treatment. Each of the apoptotic indices was calculated as the percentage of the whole PPU-7 population. Values are the mean percentage ± standard error (* *p* < 0.01, Steel’s test). No Laser: control without LI.

Apoptotic bodies were observed in hematoxylin-eosin (HE)-stained sections of PPU-7 cells exposed to Er:YAG-LI, diode-LI, or no LI (control) (Figure 2). Eosinophilic apoptotic bodies in the HE-stained PPU-7 sections, detected by light microscopy on days 1 and 3, are shown in Figure 2A,B, respectively. The same PPU-7 wells were used for an immunohistochemical cleaved caspase-3 assay (CASP3 in Figure 2A,B). In contrast to the negative controls (NC in Figure 2A,B), putative pre-apoptotic cells were observed, which were characterized by a brown antibody stain primarily in the cytoplasm. We further quantitated the occurrence of cleaved caspase-3-positive cells. The total number of caspase-3-positive apoptotic events counted for three groups, and the apoptotic indices (AIs) calculated for the treatment groups are shown in Figure 2C. In the control, less than 6% of the cells exhibited detectable caspase-3 (5.43 ± 0.73% on day 1 and 4.01 ± 0.45% on day 3). AIs in the Er:YAG laser-treated PPU-7 were 8.81 ± 0.82% on day 1, and 14.2 ± 1.03% on day 3, whereas the diode laser-treated PPU-7 cells had an AI of 8.51 ± 0.76% on day 1 and 6.81 ± 0.51% on day 3. AIs in both LI groups were significantly higher than in the control (approximately 1.63-fold on day 1 and 3.53-fold on day 3 for the Er:YAG laser, and 1.57-fold on day 1 and 1.70-fold on day 3 for the diode laser).

### 2.3. Effect of LI on Differentiation and Gene Expression in PPU-7

**Figure 3:**
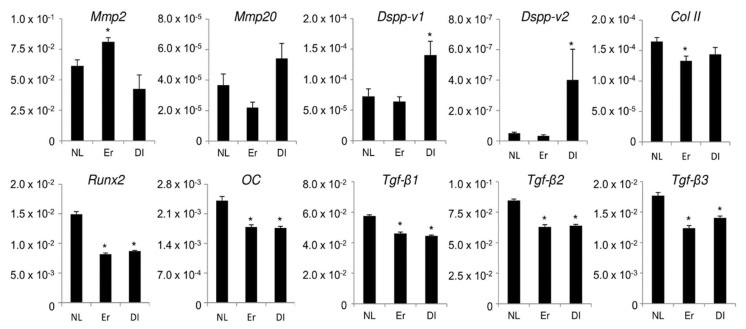
Effect of LI on gene expression in PPU-7. The mRNA expression assessed by qPCR analysis of MMP2 (*Mmp2*), MMP20 (*Mmp20*), DSPP-variant 1 (*Dspp-v1*), and DSPP-variant 2 (*Dspp-v2*) as odontoblastic differentiation markers; osteocalcin (*OC*) and runt-related transcription factor 2 (*Runx2*) as osteoblastic differentiation markers; type II collagen (*Col II*) as a chondrogenic differentiation marker; and three TGF-β isoforms (*Tgf-β1, Tgf-β2* and *Tgf-β3*). Each ratio was normalized using glyceraldehyde-3-phosphate dehydrogenase (*Gapdh*) as the reference gene, and the relative abundance of *Dspp-v1*, *Dspp-v2*, *Mmp2*, *Mmp20*, *OC*, *Runx2*, *Col II*, *Tgf-β1*, *Tgf-β2*, and *Tgf-β3* in PPU-7 was generated based on a mathematical model for relative quantification in a qPCR system. Values are the means ± standard error of 6 culture wells. The asterisk (*) on the bar graph indicates a significant difference (* *p* < 0.05, Mann–Whitney *U* test) between samples with and without LI. NL: no LI; Er: Er:YAG-LI; DI: diode-LI.

We next investigated the effect of LI on gene expression in PPU-7. The gene expression of a panel of odontoblastic, osteoblastic, and chondrocytic markers in PPU-7 on day 3 following LI was analyzed using qPCR (Figure 3). We quantified the mRNA expression of the odontoblastic differentiation markers matrix metalloproteases 2 (*Mmp2*) and 20 (*Mmp20*), and two products from the full-length dentin sialophosphoprotein (DSPP) transcript: a segment containing both the dentin glycoprotein and dentin phosphoprotein (DGP+DPP) coding regions (*Dspp-v1*), and a smaller segment specific for the dentin sialoprotein (DSP)-only transcript (*Dspp-v2*). The expression of *Mmp20*, *Dspp-v1*, and *Dspp-v2* significantly increased compared with that in the control (no LI) under diode-LI by 1.48-fold for *Mmp20*, 1.93-fold for *Dspp-v1* and 16.2-fold for *Dspp-v2*. In contrast, *Mmp2* mRNA significantly increased after Er:YAG-LI to 1.32-fold higher than the control. We also amplified runt-related transcription factor 2 (*Runx2*) and osteocalcin (*OC*) as osteoblastic differentiation markers and type II collagen (*Col II*) as a chondrocytic differentiation marker. mRNA levels significantly decreased after both LIs, Er:YAG-LI (0.74-fold for *OC*, 0.55-fold for *Runx2* and 0.81-fold for *Col II*) and diode-LI (0.73-fold for *OC*, 0.58-fold for *Runx2* and 0.87-fold for *Col II*), compared with those in the control. Moreover, the two LIs affected the expression of TGF-β isoforms, which was significantly reduced (Er:YAG-LI: 0.80-fold for *Tgf-β1*, 0.74-fold for *Tgf-β2* and 0.70-fold for *Tgf-β3*; diode-LI: 0.77-fold for *Tgf-β1*, 0.76-fold for *Tgf-β2* and 0.79-fold for *Tgf-β3*) compared with that in the control.

**Figure 4:**
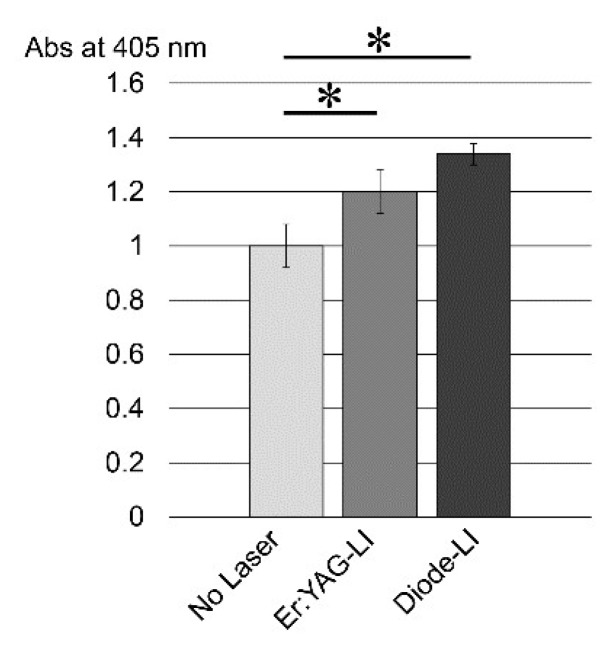
Effect of LI on ALP activity in PPU-7. ALP-inducing activity in PPU-7 after 3 days of exposure to a Er:YAG laser (Er:YAG-LI) or a diode laser (Diode-LI) (*n* = 6). No Laser: control without LI. Values are the mean ± standard error (* *p* < 0.01, Steel’s test).

Since ALP is known to be a key differentiation marker for identifying mesenchymal cells, we further investigated the effects of LI on PPU-7 ALP activity (Figure 4). The control ALP activity on day 3 was 1.0, whereas both LIs significantly enhanced inherent ALP activity in PPU-7 cells (approximately 1.20-fold for Er:YAG-LI and 1.33-fold for diode-LI).

### 2.4. Effect of LI on Mineralization Induction in PPU-7

**Figure 5:**
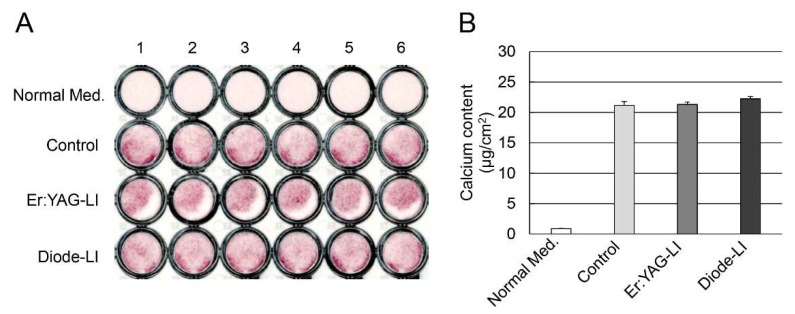
Effect of LI on mineralization nodule formation in PPU-7. (**A**) Nodule cultures with and without mineralization induction were stained with 1% Alizarin Red S on day 7. Nodule formation was apparent in PPU-7 after the two laser treatments (Er:YAG-LI and diode-LI), in contrast to cells not induced for mineralization (Normal Med.). Mineralization nodules in PPU-7 also formed under mineralization induction conditions without LI (Control). (**B**) The calcium content of PPU-7 was determined on day 7 after mineralization induction. Values are the means ± standard error of 6 culture wells.

To examine the effect of LIs on mineralization inducibility, we cultured PPU-7 in a mineralization-inducing culture medium (Figure 5), and nodule formation and mineralization capacity were assessed with Alizarin Red S staining (Figure 5A). On day 7 following the mineralization induction, precipitated nodules were evident in plates with Alizarin Red S staining, in contrast to control cells not induced for mineralization. Interestingly, the semi-cylindrical peeled portion was observed in all wells (*n* = 6) of Er:YAG-LI.

We also quantitatively analyzed PPU-7 calcium content (Figure 5B). On day 7 following mineralization induction, there were no significant differences among the control, Er:YAG-LI, and diode-LI.

### 2.5. Effect of LI on Protease Activation in Dental Pulp Tissue

**Figure 6:**
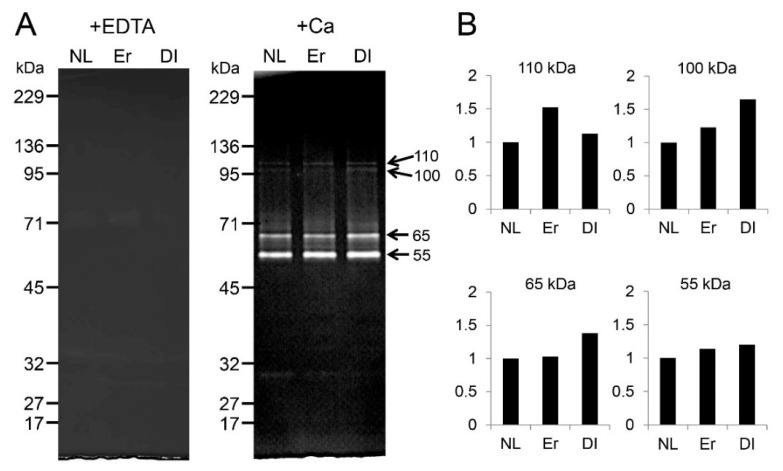
Effect of LI on porcine dental pulp tissues. (**A**) Zymogram using a gelatine gel as the substrate incubated with EDTA (left) or Ca^2+^ (right). Ten micrograms of protein extracted from porcine dental pulp tissues were used for zymography. (**B**) Densitometry analysis of 110 kDa, 100 kDa, 65 kDa, and 55 kDa protease bands. The intensity of the protease bands on the gelatin zymogel was determined using ImageJ software and normalized to the intensity of NL to compare the effect of LI. NL: without LI; Er: Er:YAG-LI; Dl: diode-LI.

In addition to the above cell-based experiments, we investigated the effect of LI on dental pulp tissues. Following LI, the minced pulp tissues were incubated with Ca^2+^ or EDTA, and extracted with Tris-guanidine buffer; the soluble fraction was analyzed by zymography (Figure 6). Protease activity was barely detected in a gelatin zymogel incubated with EDTA (Figure 6A, left). After gelatin zymography incubation with Ca, LI enhanced the intensity of the four proteases, which have molecular weights of approximately 110 kDa, 100 kDa, 65 kDa, and 55 kDa (Figure 6A, right). Figure 6B shows the densitometry analysis of the four protease bands. The intensity level of the control (no LI) was set to 1.0, and the intensity of each protease band after Er:YAG-LI increased 1.52-fold for 110 kDa, 1.23-fold for 100 kDa, and 1.14-fold for 55 kDa. There was little change between the control and Er:YAG-LI for the 65 kDa protease band. Likewise, the intensity level of each protease band after diode-LI increased 1.13-fold for 110 kDa, 1.65-fold for 100 kDa, 1.38-fold for 65 kDa, and 1.20-fold for 55 kDa.

### 2.6. Effect of LI on TGF-β Activation

**Figure 7:**
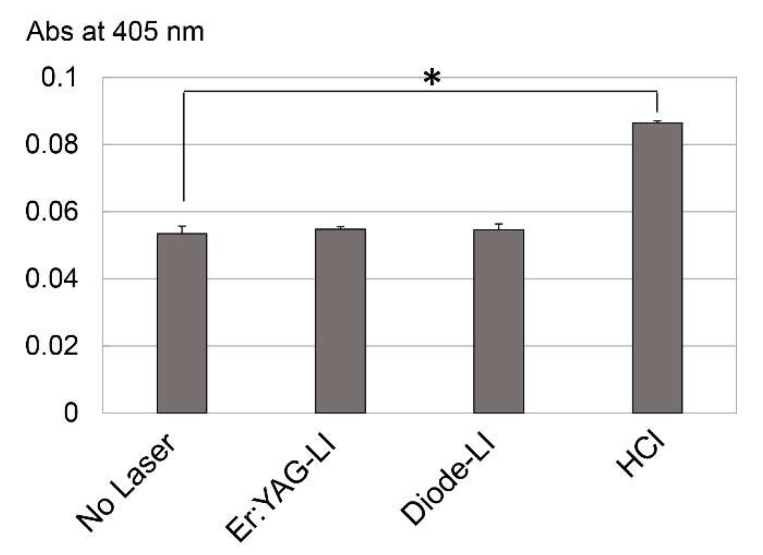
In vitro activation of latent TGF-β1 by Er:YAG-LI and diode-LI. ALP-inducing activity of HPDL cells after 3 of exposure to Er:YAG laser (Er:YAG-LI), diode laser (Diode-LI), and HCl treatment (HCl) (*n* = 6). No Laser: control without LI. Values are the mean ± standard error (* *p* < 0.05, Steel’s test).

Since we previously observed that MMP2 and MMP11 activate TGF-β1 in porcine dental pulp tissues and odontoblasts [34], we further attempted to directly activate recombinant human-latent TGF-β1 (rh-latent TGF-β1) with Er:YAG and diode lasers by measuring ALP activity in human periodontal ligament (HPDL) cells (see Appendix A.5 in Appendix A) (Figure 7). Following the laser irradiations and HCl treatment (positive control), we added the TGF-β1 sample to the HPDL cells and measured ALP activity after 3 days of incubation. Although the rh-latent TGF-β1 was activated by HCl, neither laser irradiations affected rh-latent TGF-β1 activation.

## 3. Discussion

We have, so far, characterized the dentin non-collagenous proteins and the dynamics of TGF-β in dentin–pulp complex using pigs as an experimental model. In order to take advantage of their information, we used the same animal model for the present study.

**Figure A4:**
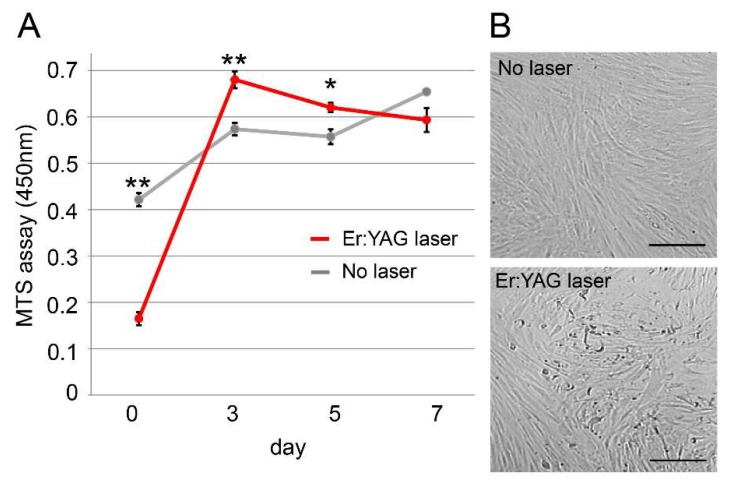
Effect of Er:YAG-LI on HPDL cells proliferation incubated for different periods [41]. (**A**) MTS (3-(4,5,dimethylthiazol-2-yl)-5-(3-carboxymethoxy-phenyl)-2-(4-sulfophenyl)-2*H*-tetrazolium, inner salt) assay. HPDL after Er:YAG-LI (red) and without LI (grey) on day 0, 3, 5, and 7 were cultured at a final volume of 120 μL/well for 1 h at 37 °C. MTS reagent was added, and an absorbance of 450 nm was recorded using a microplate reader (*n* = 10 tests per sample). Values are the mean ± standard error (* *p* < 0.05 and ** *p* < 0.01, Mann–Whitney *U* test). (**B**) Morphological changes of HPDL cells at day 2 following Er:YAG-LI. (scale bar = 200 μm).

Low-power laser irradiation (LPLI) promotes proliferation by cellular stimulating signal pathways [35,36], and delaying cell differentiation by inducing cell activation and proliferation [37]. Low-power Er:YAG-LI promotes osteoblast proliferation via the activation of MAPK/ERK in a mouse-derived osteoblastic cell line [38] and increases cell proliferation, differential protein expression, and ALP activity in human gingival fibroblasts [39,40] and HPDL cells [41]. Low-power diode-LI also boosts proliferation in human dental pulp-derived fibroblast-like cells [42] and in human cervical cancer-derived cells (HeLa cells) [43]. Using human periodontal ligament (HPDL) cells, we previously showed that Er:YAG-LI significantly increased the cell growth at day 3 (Figure A4) [41]. However, this result led us to investigate the cell growth rate up to day 3 (i.e., day 1 and day 2) following the laser irradiation. In the present study using PPU-7 cell line, we demonstrated that the cell growth rate after Er:YAG-LI was slow on day 1, and the cell population doubling time was increased. This finding suggests that Er:YAG-LI affects the cell growth right after laser irradiation. Moreover, we found that the cell growth rate after Er:YAG-LI and diode-LI was different between the two LIs on day 1, although the cell number was the almost same. This finding may suggest that part of the cell, after Er:YAG-LI, is moving towards apoptosis, besides the cell growth. Thus, we demonstrated that both Er:YAG-LI and diode-LI promoted proliferation in PPU-7, although the cell growth rate up to day 3 was different between the two LIs. This finding suggests that each LI may affect metabolic rate early in cell differentiation, in addition to the difference in the irradiation period, time, and output of both lasers.

In addition to promoting proliferation, high-fluence LPLI inhibits cell viability and induces apoptosis [35,44,45]. It has been shown that low-power HeNe irradiation induces apoptosis by accelerating Ca^2+^ uptake into the mitochondria [46]. Both histopathologic and proteomic analyses have revealed that Er:YAG-LI causes apoptosis in the skin [47]. Diode-LI has also been shown to induce apoptosis in human retinal pigment epithelial [48]. We previously showed that the morphology of HPDL cells at day 2 following Er:YAG-LI possessed the irregular forms compared to that of control (i.e., no laser irradiation) (Figure A4) [41]. This result led us to investigate apoptosis study in detail. In the present study, although the rate of apoptosis induced by laser irradiation varies by laser type, output, irradiation distance, and irradiation time, our in vitro study demonstrated that Er:YAG-LI and diode-LI for PPU-7 cause approximately 14.2% and 6.8% apoptosis, respectively, at day 3. Our results suggest that each LI has distinct effects on intrinsic apoptotic pathways via mitochondrial changes.

In general, caspase-3 plays a key role in the execution phase of cell apoptosis via extrinsic, intrinsic, or perforin/granzyme pathways [49,50]. Surprisingly, caspase-3 upregulates compensatory cell growth by inducing the production of prostaglandin E_2_ [51,52]. In fact, we demonstrated that PPU-7 cells were able to proliferate, although the rates of cell growth and apoptosis on days 1 and 3 following LI varied, depending upon the laser characteristics. This suggests that VPT using a laser helps dental pulp cells adapt to an environment of harmful side-effects, such as apoptosis.

In rat molars, diode-LI increased dentin matrix protein 1 (DMP1) and osteopontin mRNA expression in the coronal pulp, followed by the formation of reparative dentin and the co-localization of DMP1 and osteopontin immunoreactivity at the site at which this tissue first appeared [53]. We previously generated two PCR amplification products from full-length DSPP (*Dspp-v1*) and DSP-only (*Dspp-v2*) transcripts. Both *Dspp-v1* and *Dspp-v2* products are predominantly expressed in odontoblasts, with only trace expression of the *Dspp-v1* transcript detected in dental pulp, and *Dspp-v2* transcript is predominantly expressed in dental pulp [54]. This study demonstrated that diode-LI enhanced the expression of both *Dspp* mRNAs, especially *Dspp-v2*, but Er:YAG-LI did not contribute substantially. Our findings suggest that diode-LI have more dominant potency to induce the gene expression of dentin non-collagenous proteins.

In addition to the expression of dentin non-collagenous proteins, MMP mRNA is also expressed in pulp and odontoblasts, where MMPs play an important role in dentin matrix formation. MMP2, MMP3, MMP8, MMP9, MMP14, and MMP20 are the main MMPs that have been identified in pulp, odontoblasts, and predentin/dentin [55,56,57,58,59,60]. Our previous study showed that *Mmp11*, and both *Mmp2* and *Mmp20* mRNAs, are predominantly expressed in porcine dental pulp tissues and odontoblasts, respectively [34]. In this study, we demonstrated that Er:YAG-LI enhanced *Mmp2* mRNA in PPU-7 cells, whereas diode-LI increased *Mmp20* expression. Judging from the expression of the two *Dspp* mRNAs, it is likely that odontoblastic gene expression in PPU-7 is regulated by the reactivity of cells based on the specific characteristics of two lasers.

ALP is believed to be the initial marker of mesenchymal cell differentiation into hard tissue-forming cells, such as osteoblasts or odontoblasts [61,62]. We demonstrated that both LIs enhanced ALP activity in PPU-7. Considering that both LIs reduced the expression of osteoblastic and chondrocytic marker genes, our findings suggest that LI induces the differentiation of PPU-7 into odontoblast-like cells.

Alizarin Red S staining has been widely used to evaluate calcium deposits in cell cultures [63]. We found that Er:YAG-LI was likely to damage a portion of the cell. When we repeated this experiment with the Er:YAG laser (*n* = 5), the same phenomenon was observed (data not shown). It is currently unknown if this damage was caused by human error during the laser treatment or by characteristics of the laser (i.e., the Er:YAG laser is absorbed on the tissue surface, whereas the diode laser is transmitted to the internal tissue). Interestingly, we also demonstrated that there were few differences in the amount of calcium deposits between the two LIs. Although both LIs exhibited minimal effect on mineralization induction, our data suggest that Er:YAG-LI might cause transient or persistent cell shrinkage.

**Figure A5:**
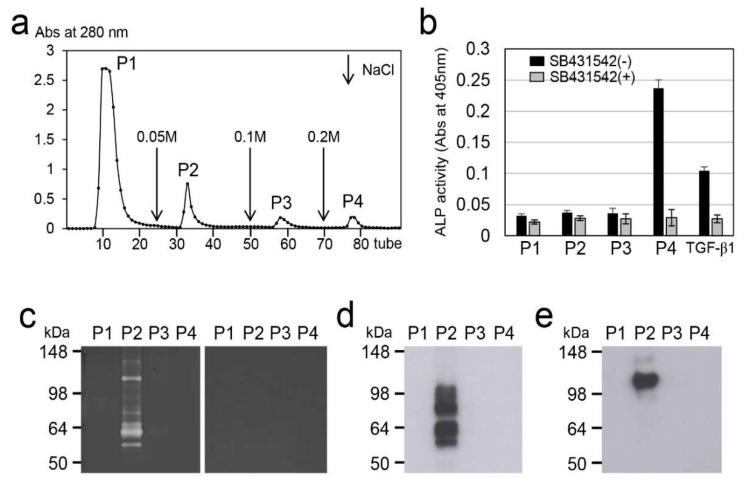
Isolation and detection of active forms of TGF-β, MMP2 and MMP11 in porcine dental pulp [33]. (**a**) Heparin-sepharose chromatogram of extracts from porcine dental pulp detected at 280 nm absorbance. Downward-pointing arrows represent the starting points of the step gradient with 0.05, 0.1, and 0.2 M NaCl. (**b**) ALP-inducing activity of HPDL cells with (+) or without (−) the addition of SB431542 to fractions P1–P4 isolated by chromatography. Recombinant human TGF-β1 with a carrier (0.3 ng/mL) (TGF-β1) was used as a positive control for the detection of ALP-inducing activity in HPDL cells. The data show the average value of six measurements. (**c**) A gelatin zymogram of fractions P1–P4 incubated without (left) or with (right) EDTA. (**d**,**e**) Western blots of fractions P1–P4 with specific antibodies against (**d**) MMP2 and (**e**) MMP11.

**Figure A6:**
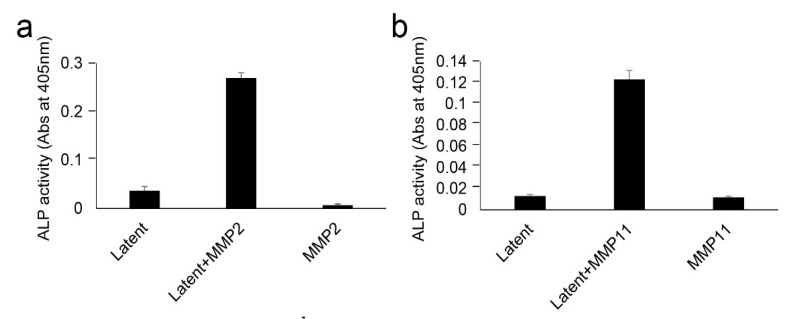
In vitro activation of latent TGF-β1 by MMP2 or MMP11 [33]. Latent TGF-β1 was incubated with rhMMP2 or rhMMP11. ALP-inducing activity of HPDL cells exposed to (**a**) latent TGF-β1 only (Latent), latent TGF-β1 with rh-MMP2 (Latent+MMP2), and rh-MMP2 only (MMP2) samples, and ALP-inducing activity of HPDL cells exposed to (**b**) latent TGF-β1 only (Latent), latent TGF-β1 with rh-MMP11 (Latent+MMP11) and rh-MMP11 only (MMP11) samples (*n* = 6).

**Figure A7:**
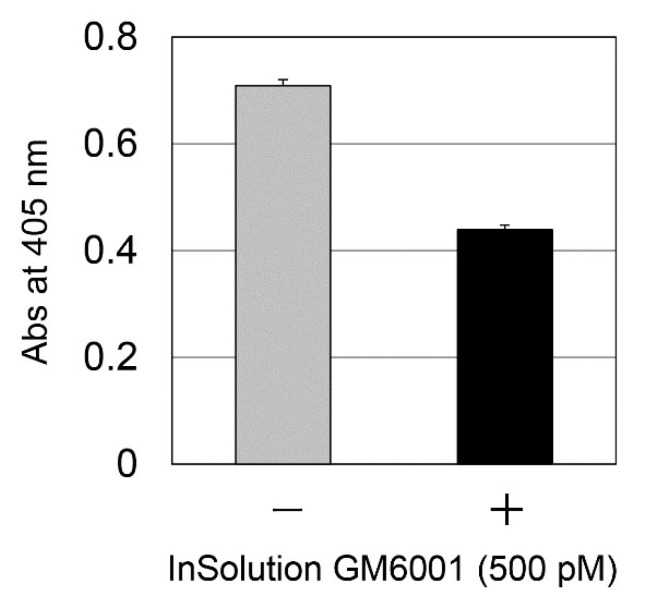
In vitro activation of endogenous TGF-β1 in HPDL cells cultured with or without MMP2 inhibitor after Er:YAG-LI. HPDL cells were incubated without (−) or with (+) 500 pM of InSolution GM6001 (MMP2 inhibitor)(Calbiochem/Merck Millipore, Darmstadt, Germany) for 5 days after Er:YAG-LI. Endogenous TGF-β activity in HPDL cells was evaluated by determining ALP-inducing activity in HPDL cells (*n* = 6).

TGF-β is a potent regulator of cell growth, cell differentiation, and extracellular matrix deposition [64]. There are three TGF-β isoforms (TGF-β1, TGF-β2 and TGF-β3) in mammals, which stimulate matrix secretion in odontoblasts, are mitogenic to pulp cells, and possess a potential inductive effect for dental pulp cell differentiation [65]. We previously separated protein samples obtained from dental pulp tissue into four fractions. TGF-β1 activity, in vivo, was detected in the fourth fraction using the ALP-HPDL system (Figure A5). TGF-β was activated by acid [66], and MMP proteolytic degradation of the latent TGF-β complex [67,68]. Our previous study showed that the active forms of two MMPs (MMP2 and MMP11) are present in dental pulp tissues (Figure A5). Moreover, our previous in vitro study showed that the incubation of rh-latent TGF-β1, without rhMMP2 or rhMMP11, exhibited only trace levels of ALP-inducing activity, whereas treatment with rhMMP2 or rhMMP11 enhanced the ALP-inducing activity (Figure A6), and active forms of TGF-β1 enhance the expression of *Dspp-v1*, *Dspp-v2*, and *Mmp20* genes by activated TGF-β1 [34]. In this study, we found that both Er:YAG-LI and diode-LI were involved in the activation of four MMPs in dental pulp tissues. This finding led us to confirm that LI can activate latent TGF-β directly, or via MMP activation. Our preliminary study showed that ALP activity was inhibited when HPDL cells were cultured with MMP2 inhibitor for 5 days after Er:YAG-LI (Figure A7). This result suggests that the activated MMP2 by laser irradiation is further activated TGF-β. In addition, our previous study showed that rh-latent TGF-β1 is not directly activated by Er:YAG-LI [41] in HPDL cells. This in vitro study demonstrated that laser irradiation might not affect the activation of rh-latent TGF-β1 in HPDL cells. Considering the qPCR analysis and ALP assay shown in Figure 3 and Figure 4, our findings suggest that both LIs possess indirect differentiation-promoting effects on odontoblasts through the enhancement of odontoblastic gene expression by the activation of MMPs. Further studies are required to investigate the canonical and/or non-canonical TGF-β pathway, for better understanding how both human and porcine cells responded to both LIs in the same manner.

## 4. Methods

All of the animal experiments were approved by the Institutional Animal Care Committee and the Recombination DNA Experiment and Biosafety Committee of the Tsurumi University School of Dental Medicine. (Project identification code #1318, 1 December 2015).

### 4.1. LI of Porcine Dental Pulp Cells (PPU-7)

The cell line (PPU-7) was established from porcine dental pulp cells by our group (see Appendix A.4 in the Appendix A). PPU-7 cells were plated on a 96-well plate (Corning Inc., Corning, NY, USA) at a density of 1 × 10^4^ cells/well or on chamber slides (Matsunami Glass Ind., LTD., Osaka, Japan) at a density of 1 × 10^4^ cells/well. Cells were cultured in standard medium for 24 h. For genetic studies, subconfluent PPU-7 cells were irradiated with a Er:YAG laser (Erwin AdvErL, Morita, Kyoto, Japan) at 50 mJ (10 pps) for 10 s, or a high-power diode laser (OSADA LIGHTSURGE SQUARE5, Osada, Tokyo, Japan) with a 1W continuous wave for 10 s on a 96 well plate. For immunohistochemical studies, subconfluent PPU-7 cells were irradiated with an Er:YAG laser at 50 mJ (10 pps) for 30 s or a high-power diode laser with a 1W continuous wave for 30 s on the chamber slide. All laser irradiations on PPU7 cells were carried out without medium, at a distance of 2 cm, in sweeping motion. PPU-7 cells not exposed to LI were used as a control. PPU-7 cells were incubated for 1 or 3 days, and characterized by MTS assay, apoptosis detection, qPCR, and ALP assay.

### 4.2. Cell Proliferation Assay

The cells were plated on 96-well plates (*n* = 6) at a density of 1500 cells/well in standard medium, and cultured at 37 °C in a humidified 5% CO_2_ atmosphere. The culture medium was changed every other day. The proliferation rate of the cells on six 96-well plates was determined on days 1, 2, 3, and 5 using a CellTiter 96^®^AQuous One Solution Cell Proliferation Assay (MTS assay) (Promega Corporation, Madison, WI, USA).

### 4.3. Assessment of Apoptosis by Immunohistochemistry

On day 1 and 3 after LI, PPU-7 cells on chamber slides were fixed with 4% paraformaldehyde for 15 min at room temperature and incubated in a blocking solution (1% BSA, 10% normal goat serum) for 1 h at room temperature. For primary antibody application, the dilution of anti-cleaved caspase-3 monoclonal antibody (Cell Signaling, Danvers, MA, USA) was used at 1:500, and the cells were incubated overnight at 4 °C. For secondary antibody application, diluted HRP-conjugated goat anti-rabbit IgG H&L antibody (abcam, Cambridge, UK) was used at 1:500, and the cells were incubated for 1 h at room temperature. The positive signal was detected using 3,3-diaminobenzidine (DAB) (TaKaRa, Kusatsu, Japan) as a staining substrate. Sections were counterstained to observe clear tissue and cell morphology using hematoxylin. Light micrographs were obtained using a Canon EOS Kiss X8i (Canon, Tokyo, Japan) camera on an optical microscope (OLYMPUS BX50, Olympus, Tokyo, Japan). The number of apoptotic cells present on a chamber slide expressed as a fraction of the total number of cells, named the “apoptotic index,” was used to evaluate apoptotic state. The number of activated caspase-3-labeled apoptotic cells and bodies was calculated in 30 high power fields (HPFs; objective X400, field diameter 640 μm). The apoptotic index was calculated as the percentage of the whole PPU-7 population.

### 4.4. Quantitative Polymerase Chain Reaction (qPCR) Analysis

RNA from PPU-7 cells on day 3 after LI was extracted using a High Pure RNA Isolation Kit (Roche Diagnostics GmbH, Mannheim, Germany). After the purified total RNA (6 μL) was reverse transcribed, a reaction mixture containing SYBR Green PCR master mix (Roche), supplemented with 0.5 µM forward and reverse primers, and 2 µL cDNA as a template was made. The specific primer sets were designed using Primer-BLAST software (available online: http://www.ncbi.nlm.nih.gov/tools/primer-blast). The specific primer sets and running conditions are shown in Table A1 in the Appendix A. GAPDH was used as a reference gene. The normalization of each ratio using relative MMP quantification data (*Mmp2* and *Mmp20*), two DSPP variants (*Dspp-v1* and *Dspp-v2*), *Runx 2, OC, Col II*, and TGF-βs (*TGF-β1*, *TGF-β2*, and *TGF-β3*) in comparison to a reference gene (*Gapdh*) was generated based on a mathematical model for the relative quantification of qPCR. All of the values are represented as the means ± standard error (SEM). Statistical significance (*) was determined using a Mann–Whitney *U* test. In all cases, *p* < 0.05 was regarded as statistically significant.

### 4.5. ALP Assay

PPU-7 cells were plated on a 96 well plate at a density of 3.16 × 10^4^ cells/cm^2^, and cultured in standard medium for 24 h. The ALP activity of each well was measured as described previously [69]. The cells on day 3 after LI were washed once with phosphate buffered saline (PBS), and ALP activity was assayed using 10 mM *p*-nitrophenylphosphate as the substrate in 100 mM 2-amino-2-methyl-1,3-propanediol-HCl buffer (pH 10.0) containing 5 mM MgCl_2_ and incubated for 5 min at 37 °C. Adding 0.2 M NaOH quenched the reaction, and the absorbance at 405 nm was read on a plate reader.

### 4.6. Formation of Precipitated Nodules in PPU-7 Cells

PPU-7 cells were grown on a 48 well plate at an initial density of 3.16 × 10^4^ cells/cm^2^ and cultured in standard medium for 24 h. Following LI, the medium was changed to mineralization-inducing medium containing 10 mM β-glycerophosphate and 50 μM ascorbic acid. The cells were further cultured up to day 7, and mineralization was visualized using Alizarin Red S staining. After fixation with a 4% paraformaldehyde neutral buffer solution for 30 min, the cells were stained with 1% Alizarin Red S (Sigma-Aldrich, St. Louis, MO, USA) solution for 10 min, washed with distilled water, and photographed. Additionally, PPU-7 cells were grown on a 48-well plate at an initial density of 3.16 × 10^4^ cells/cm^2^. After incubation for 24 h, the medium was changed to mineralization medium, and the cells were cultured up to day 7. Each well on the plates was rinsed with PBS, and Ca^2+^ was dissolved in 0.2 mL of 0.5 N HCl by gentle rocking for 1 h. The calcium concentration in the eluate was spectrophotometrically determined at 570 nm by following color development with a Ca^2+^ assay kit (Calcium C-Test Wako, Wako Pure Chemical Industries, Ltd., Osaka, Japan). All of the values were normalized against the cultivation area.

### 4.7. LI of Porcine Dental Pulp Tissues and Protein Extraction

Tooth germs of permanent molars were surgically extracted from the mandible of deceased 5-month-old pigs (*n* = 10) within one hour after slaughter from the Meat Market of the Metropolitan Central Wholesale Market (Shinagawa, Tokyo, Japan). Pulp tissue (17 g) pulled from tooth germs was briefly rinsed in ice-cold sterile PBS to remove blood cells, and minced with a surgical blade. Minced pulp tissue (1 g each) was irradiated with an Er:YAG laser at 50 mJ (10 pps) for 60 s or a high-power diode laser with a 1W continuous wave for 60 s on a petri dish. The pulp sample was transferred into a conical tube (15 mL) and incubated in 5 mL of 50 mM Tris-HCl buffer (pH 7.4) containing 10 mM CaCl_2_ or 10 mM EDTA for 20 h at 37 °C. Following incubation, the buffer was removed by centrifugation, and the pulp tissues were suspended in 50 mM Tris-HCl/4 M guanidine buffer (pH 7.4) containing protease inhibitor cocktail (23 mM 4-(2-aminoethyl) benzenesulfonyl fluoride hydrochloride (AEBSF), 100 mM EDTA, 2 mM bestatin, 0.3 mM E-64 and 0.3 mM pepstatin A (Sigma-Aldrich)) and homogenized using a PHYSCOTRON micro-homogenizer (MICROTEC, Co., Ltd., Funabashi, Chiba, Japan), for 30 s at half speed. Insoluble material was pelleted by centrifugation (15,900 g). The supernatant was desalted with an Amicon Ultra centrifugal filter (0.5 mL, MW = 3000 cut off) (Merck Millipore, Darmstadt, Germany) and characterized by SDS-PAGE and zymography.

### 4.8. Zymography

Zymography was performed using Novex 10% Zymogram Gelatin Gel (Life Technologies/Invitrogen/Thermo Fisher Scientific, Waltham, MA, USA) or Precast Casein Zymogram Gel (Cosmo Bio Co., LTD., Tokyo, Japan). Samples were dissolved in NuPAGE LDS sample buffer (Invitrogen), and electrophoresis was performed at 30 mA for approximately 1 h with Novex Tris Glycine SDS running buffer (Life Technologies/Invitrogen/Thermo Fisher Scientific). The gel was shaken gently in 2.5% Triton X-100 solution for 1 h at room temperature with one buffer change, and incubated overnight with or without 10 mM EDTA in 50 mM Tris–HCl (pH 7.4) containing 10 mM CaCl_2_. Proteinase activities were visualized as unstained bands after the gel was stained with Coomassie Brilliant Blue R-250 (CBB) (Bio-Rad Laboratories, Hercules, CA, USA). The apparent molecular weights of the protein bands were estimated by comparison with DynaMarker Protein MultiColor III (BioDynamics Laboratory Inc., Tokyo, Japan).

### 4.9. In Vitro Activation of Latent TGF-β1 by LI

Recombinant human latent TGF-β1 (rh-latent TGF-β1, Cell Signaling Technology, Danvers, MA, USA) was added to a 96 well plate at a concentration of 125 ng/25 μL, and irradiated with an Er:YAG laser at 50 mJ (10 pps) for 10 s, or a high-power diode laser with a 1W continuous wave for 10 s. In addition, rh-latent TGF-β1 (100 ng/20 μL) was incubated with 20 μL of 0.1 N HCl for 1 h at room temperature in a microtube. Following laser irradiation or HCl treatment, rh-latent TGF-β1 was added to subconfluent HPDL cells (1.0 × 10^4^ cells/well on 96 well plate) at a final concentration of 3.2 ng/mL. Following incubation for 3 days, each reaction sample was characterized using the ALP-HPDL system (see Appendix A.5 in the Appendix A).

### 4.10. Statistical Analysis

For the MTS and ALP assays, AIs, and qPCR and calcium analyses, all of the values are represented as the means ± standard errors. Statistical significance (*) was determined using a Mann–Whitney *U* test for qPCR analysis and a nonparametric Steel’s test for the MTS and ALP assays and the AIs and calcium analysis. In all of the tests, *p* < 0.01 for MTS and ALP assays and *p* < 0.05 for AIs, qPCR and calcium analyses were regarded as statistically significant.

## 5. Conclusions

**Figure 8:**
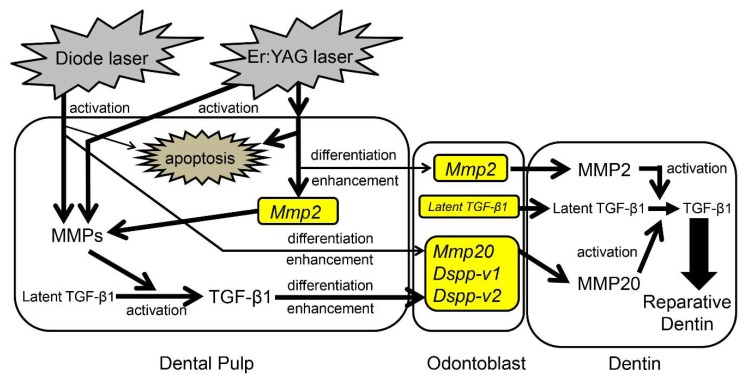
Proposed biological reaction mechanism in porcine dental pulp cells and tissues induced by Er:YAG-LI and diode-LI.

During dental treatment with lasers, it is necessary to understand the mechanism of reparative dentin formation. We summarize the potential biological reaction mechanism of the cellular response to Er:YAG and diode lasers in porcine dental pulp cells and tissues in Figure 8.

Beside apoptosis, both Er:YAG and diode lasers activate some MMPs in dental pulp. Alternatively, Er:YAG laser enhances the expression and presumably production of *Mmp2*. Activated MMPs are further involved in the activation of latent TGF-β1, followed by the differentiation of odontoblasts and the enhancement of odontoblastic gene expression by activated TGF-β1. Moreover, both lasers promote the differentiation of odontoblasts and the expression of odontoblastic-marker genes through other factors. In endodontic therapy, the present study helped elucidate how reparative dentin is formed by laser treatment for pulp exposure. Further studies are required to understand TGF-β signaling downstream molecules (i.e., canonical and/or non-canonical TGF-β pathway) and elucidate the morphological and histological efficacy of lasers in animal experiments. Moreover, it is necessary to expand the experiments with human cells.

## Abbreviations

MTS	3-[4,5,dimethylthiazol-2-yl]-5-[3-carboxymethoxy-phenyl]-2-[4-sulfophenyl]-2*H*-tetrazolium inner
mRNA	messenger ribonucleic acid
qPCR	quantitative polymerase chain reaction
EDTA	ethylenediaminetetraacetic acid
SDS	sodium dodecyl sulfate
PAGE	polyacrylamide gel electrophoresis
kDa	kilodalton
HeNe	helium-neon
HPFs	high-power fields
GAPDH	glyceraldehyde-3-phosphate dehydrogenase

## Appendix A

### Appendix A.1. Immortalization of Porcine Pulp Cells and ALP Expression

We established porcine dental pulp-derived cell lines using the porcine animal model. Following the transfection with pSV3-neo plasmid, we incubated over 20 G418-resistant colonies. However, as most of the cell growth was stopped, we selected 8 clones (PPU-1, -3, -7, -10, -12, -16, -17, and -18). The immortalized pulp cells’ morphology was fibroblast-like (Figure A1A).

Since alkaline phosphatase (ALP) has been believed to be the initial marker for the differentiation of mesenchymal cells into hard tissue-forming cells, such as osteoblasts or odontoblasts [61,62], we determined the inherent ALP activity of the eight immortalized pulp cell lines. Both PPU-3 and PPU-7 cell lines possessed high inherent ALP activity, whereas the PPU-16 cell line had a trace of ALP activity (Figure A1B). We further investigated the effects of BMP2 and TGF-β1 on ALP activity of eight immortalized pulp cells along with the determination of inherent ALP activity. The addition of BMP2 enhanced an increasing ratio of ALP activity in PPU-10 (~1.5-fold) and PPU-16 (~2.2-fold) cell lines, when the activity of control (i.e., when eight immortalized pulp cells were cultured without any cytokines) was 1 (Figure A2A). When we cultured those two cell lines with BMP2 and LDN-193189, which is a selective BMP signaling inhibitor, the ALP activity level was decreased to below baseline. ALP activity of other cell lines (PPU-1, -3, -7, -12, -17, and -18) with the addition of BMP2 was almost baseline levels. Although PPU-16 possessed the lowest ALP activity of the eight clones, it showed the highest increase in ALP activity in response to BMP2. In contrast, the addition of TGF-β1 decreased ALP activity in each pulp cell below baseline (Figure A2B). When we cultured each pulp cell with TGF-β1 and SB431542, which is a specific and selective inhibitor of TGF-β type I activating receptor-like kinase (ALK) receptors, the ALP activity was restored to near baseline levels. Based on these experimental results, we selected two cell lines: PPU-7, possessing the high inherent ALP activity, and PPU-16, showing the high reactivity for BMP2.

### Appendix A.2. Cell Proliferation Rate

We investigated the effects of BMP2 and TGF-β1 on cell proliferation in the PPU-7 and PPU-16 cell lines and expressed cell density using a semi-logarithmic scale to judge the logarithmic growth phase by linearity (Figure A3A). TGF-β1 treatment inhibited the cell proliferation in both PPU-7 and PPU-16 cell lines, whereas BMP2 treatment inhibited the cell proliferation in PPU-7 cell line only. No significant differences were observed between the control and BMP2 treatment in the PPU-16 cell line. Compared with the control, the cell population doubling time of PPU-7 increased significantly with BMP2 or TGF-β1 treatment (Figure A3B). TGF-β1 treatment significantly increased the doubling time of the PPU-16 cell line, but BMP2 treatment did not (Figure A3B).

### Appendix A.3. Gene Expression in PPU-7 and PPU-16 Cell Lines

Gene expression of a panel of odontoblastic markers in both PPU-7 and PPU-16 cell lines at 1, 3, and 7 days following BMP2 or TGF-β1 treatments was analyzed by qPCR (Figure A3C). We amplified two products from the full-length *Dspp* transcript: a segment containing the DGP+DPP coding region (DSPP-v1), and a smaller segment specific for the DSP-only transcript (DSPP-v2). In addition to those dentin markers, we checked the expression of ALP as the initial marker for the differentiation of dental pulp cells. Expression levels of DSPP-v1, DSPP-v2, and ALP in PPU-7 cell line were higher than those in PPU-16 cell line throughout the culture period. Comparing the effects of BMP2 and TGF-β1, both BMP2 and TGF-β1 treatments upregulated both DSPP-v1 and DSPP-v2 expression levels in PPU-7 cell line at 7 days. Both BMP2 and TGF-β1 treatments also gradually upregulated ALP expression level in the PPU-7 cell line, but TGF-β1 treatment did not affect the PPU-16 cell line. Thus, based upon all the above results, we selected PPU-7 cell line for the present study.

### Appendix A.4. Cell Line Isolation, Transfection, and Establishment from Porcine Dental Pulp Cells

Tooth germs of permanent incisors were surgically extracted from the mandibles of deceased 5-month-old pigs (*n* = 10) within one hour after slaughter from the Meat Market of Metropolitan Central Wholesale Market (Shinagawa, Tokyo, Japan). Pulp tissue pulled out from tooth germs was briefly rinsed in ice-cold sterile PBS to remove blood cells, minced with a surgical blade, and digested in a solution of 0.1% collagenase and 0.2% dispase II (Wako Pure Chemical Industries, Osaka, Japan) for 1 h at 37 °C with gentle shaking. The released cells were passed through a 70 µm cell strainer (BD Falcon, Bedford, MA, USA) and washed three times with PBS by centrifugation. The cells were then cultured in alpha Minimum Essential Medium (αMEM) (Gibco/Life Technologies, Carlsbad, CA, USA) containing 10% fetal bovine serum (FBS) and antibiotics (50 U/mL of penicillin, 50 µg/mL of streptomycin, Gibco) at 37 °C in a humidified 5% CO_2_ atmosphere. Pulp cells isolated from porcine tooth germs were plated at subconfluent cell densities, and transfected with the pSV3-neo plasmid (ATCC 37150) using Lipofectamine 2000 (Invitrogen/Life Technologies, Carlsbad, CA, USA) in accordance with the manufacturer’s instructions. The pSV3-neo plasmid expresses the SV40 large T-antigen for immortalization and neomycin phosphotransferase for selection. Two days after transfection, the cells were re-plated at low density and selected with 0.35 mg/mL of geneticin (G418) (ATCC, Manassas, VA, USA). The cells were cultured in media containing G418 until colonies were visible. Individual colonies were isolated with cloning cylinders and maintained in the standard medium at 37 °C in a humidified 5% CO_2_ atmosphere.

### Appendix A.5. ALP-HPDL System

The human periodontal ligament (HPDL) cells were plated on a 96-well plate at a density of 3.16 × 10^4^ cells/cm^2^, and were cultured in the standard medium for 24 hrs. The medium was changed to growth medium with 50 nM of 1α,25(OH)_2_D_3_ (EMD Millipore Corp., Billerica, MA, USA). Measuring the ALP activity in each well was described previously [69]. After 72 additional hours of incubation, the cells were washed once with phosphate buffered saline (PBS), and ALP activity was assayed using 10 mM *p*-nitrophenylphosphate as the substrate in 100 mM 2-amino-2-methyl-1,3- propanediol-HCl buffer (pH 10.0) containing 5 mM MgCl_2_, and incubated for 5 min at 37 °C. After quenching with 0.2 M NaOH, the absorbance at 405 nm was read using a plate reader.

**Table A1:** Selected primers, size of amplified product for qPCR analysis shown in main Figure 3.

Gene		Sequence (5′→3′)	Size (bp)	qPCR Protocol (45 Cycles)		
*Mmp2*	F	CCGACGTGGCCAATTACAAC	96			
	R	GGTCCAGATCAGGCGTGTAG				
*Mmp20*	F	ATGCAGCTTACGAAGTGGCT	95			
	R	GGGAGGACCTTGCATTTGGA				
*Dspp-v1*	F	CCCAGAAACCCAATCAGAGA	300			
	R	TATGTGTTTTGCTGGGTCCA				
*Dspp-v2*	F	CCCAGAAACCCAATCAGAGA	149			
	R	GGGAAGGAAGGGGAGAATTT				
*Runx2*	F	CAACTTCCTGTGCTCTGTGC	119	Denaturation	95 °C	10 s
	R	CCGCCATGACAGTAACCACA				
*OC*	F	CCAGGCAGATGCAAAGCCTA	96	Annealing	60 °C	10 s
	R	CGCCTGAGTCTCTTCACCAC				
*Col II*	F	CCAGATTGAGAGCATCCGCA	142	Extension	72 °C	10 s
	R	CATGGCGTCCAAAGTGCATC				
*Tgf-β1*	F	GCCTGCTGAGGCTCAAGTTA	131			
	R	ATCAAAGGACAGCCACTCCG				
*Tgf-β2*	F	GCGCTACATCGACAGCAAAG	143			
	R	TGCAGCAGGGACAGTGTAAG				
*Tgf-β3*	F	CTGTGCGTGAATGGCTCTTG	93			
	R	TATCCCCGTTGGGCTGAAAG				
*Gapdh*	F	CCATCACCATCTTCCAGGAG	346			
	R	ACAGTCTTCTGGGTGGCAGT				
